# Elevated brain lactate in schizophrenia: a 7 T magnetic resonance spectroscopy study

**DOI:** 10.1038/tp.2016.239

**Published:** 2016-11-29

**Authors:** L M Rowland, S Pradhan, S Korenic, S A Wijtenburg, L E Hong, R A Edden, P B Barker

**Affiliations:** 1 Department of Psychiatry, Maryland Psychiatric Research Center, University of Maryland School of Medicine, Baltimore, MD, USA; 2 Department of Radiology and Radiological Sciences, Johns Hopkins University School of Medicine, Baltimore, MD, USA; 3 Department of Psychology, University of Maryland Baltimore County, Baltimore, MD, USA; 4 Kirby Imaging Center, Kennedy Krieger Institute, Baltimore, MD, USA

## Abstract

Various lines of evidence suggest that brain bioenergetics and mitochondrial function may be altered in schizophrenia. On the basis of prior phosphorus-31 (^31^P)-magnetic resonance spectroscopy (MRS), post-mortem and preclinical studies, this study was designed to test the hypothesis that abnormal glycolysis leads to elevated lactate concentrations in subjects with schizophrenia. The high sensitivity of 7 Tesla proton (^1^H)-MRS was used to measure brain lactate levels *in vivo*. Twenty-nine controls and 27 participants with schizophrenia completed the study. MRS scanning was conducted on a Philips ‘Achieva' 7T scanner, and spectra were acquired from a voxel in the anterior cingulate cortex. Patients were assessed for psychiatric symptom severity, and all participants completed the MATRICS Consensus Cognitive Battery (MCCB) and University of California, San Diego Performance-Based Skills Assessment (UPSA). The relationship between lactate, psychiatric symptom severity, MCCB and UPSA was examined. Lactate was significantly higher in patients compared with controls (*P*=0.013). Higher lactate was associated with lower MCCB (*r*=−0.36, *P*=0.01) and UPSA total scores (*r*=−0.43, *P*=0.001). We believe this is the first study to report elevated *in vivo* cerebral lactate levels in schizophrenia. Elevated lactate levels in schizophrenia may reflect increased anaerobic glycolysis possibly because of mitochondrial dysfunction. This study also suggests that altered cerebral bioenergetics contribute to cognitive and functional impairments in schizophrenia.

## Introduction

Schizophrenia is a severe mental disorder characterized by hallucinations, disorganized thought, impaired emotional and motivational processes, and cognitive dysfunction. Antipsychotic medications help diminish positive symptoms but do not alleviate negative symptoms or cognitive impairments, and therefore many people with schizophrenia continue to suffer severe functional impairment despite treatment. Traditionally, the pathophysiological mechanisms believed to be associated with schizophrenia have centered on neurotransmitter systems, in particular the dopaminergic, glutamatergic and GABAergic systems. Although less explored, it has also been proposed that mitochondrial and bioenergetic alterations may have a role in the pathophysiology of this illness, either directly or via alterations in underlying neurotransmitter systems.^1^ Post-mortem,^2, 3, 4^ preclinical,^5^ cerebrospinal fluid (CSF)^6^ and phosphorus-31 (^31^P)-magnetic resonance spectroscopy (MRS)^7^ studies have each provided some support for mitochondrial and bioenergetic abnormalities in schizophrenia. A recent ^31^P magnetization transfer MRS study^7^ of the medial frontal region that included the anterior cingulate found reduced creatine kinase reaction rate and pH in schizophrenia, which was interpreted as being consistent with dysfunctional glucose metabolism and the accumulation of lactate. This interpretation was supported by prior reports of increased lactate levels in CSF^6^ and post-mortem brain tissue^3^ in schizophrenia.

However, to date, there are no reports, that we are aware of, on the measurement of brain lactate levels in patients with schizophrenia *in vivo*. In the normal brain, lactate is present at low quantities and is barely detectable using conventional MRS at widely available field strengths of 1.5 or 3.0 T. Therefore, this study assessed brain lactate levels using the higher sensitivity of high field (7 T) MRS coupled with 32-channel receiver coils,^8, 9, 10^ in both participants with schizophrenia and healthy control participants. The measured region was similar to that defined in a recent ^31^P magnetization transfer MRS study.^7^ It was hypothesized that lactate levels would be elevated and related to poor cognitive function and severity of symptoms in schizophrenia.

## Materials and methods

Thirty-one controls and thirty participants with schizophrenia took part in this study. Patients were recruited from the Maryland Psychiatric Research Center outpatient clinics and neighboring mental health clinics. Community controls were recruited through media advertisements and random digit dialing targeting the same community where our patients reside. Participants with schizophrenia had a diagnosis of schizophrenia or schizoaffective disorder as determined with the Structured Clinical Interview for Diagnostic and Statistical Manual of Mental Disorders, Fourth Edition, Text Revision, Patient Version.^11^ Control participants had no past or present Axis I psychiatric disorder as determined with the Structured Clinical Interview for DSM-IV-TR.^11^ All participants were 18–55 years old, with no current or past neurological condition and major medical conditions, and no DSM-IV-TR substance abuse in the last 6 months or substance dependence in lifetime. All but five participants with schizophrenia were treated with antipsychotic medication, and dosages were converted to chlorpromazine (CPZ) equivalents.^12^ One person was taking depakote and none were taking benzodiazepines, or anticholinergics. Participants with schizophrenia were evaluated for their capacity to provide informed consent before signing consent documents. All participants gave written informed consent before participation in the study. This study was approved by the University of Maryland Baltimore and Johns Hopkins Medicine Institutional Review Boards.

Patients were evaluated for psychopathology with the Brief Psychiatric Rating Scale^13^ and the Brief Negative Symptom Scale.^14^ Both patient and control participants completed the MATRICS Consensus Cognitive Battery (MCCB)^15^ as a measure of general cognitive function, and the University of California, San Diego Performance-based Skills Assessment (UPSA-2)^16^ for assessment of functional capacity across five domains: organization/planning, financial skills, communication skills, transportation and household skills. Participants were monetarily compensated for their time.

### MRS acquisition and analyses

**Figure 1:**
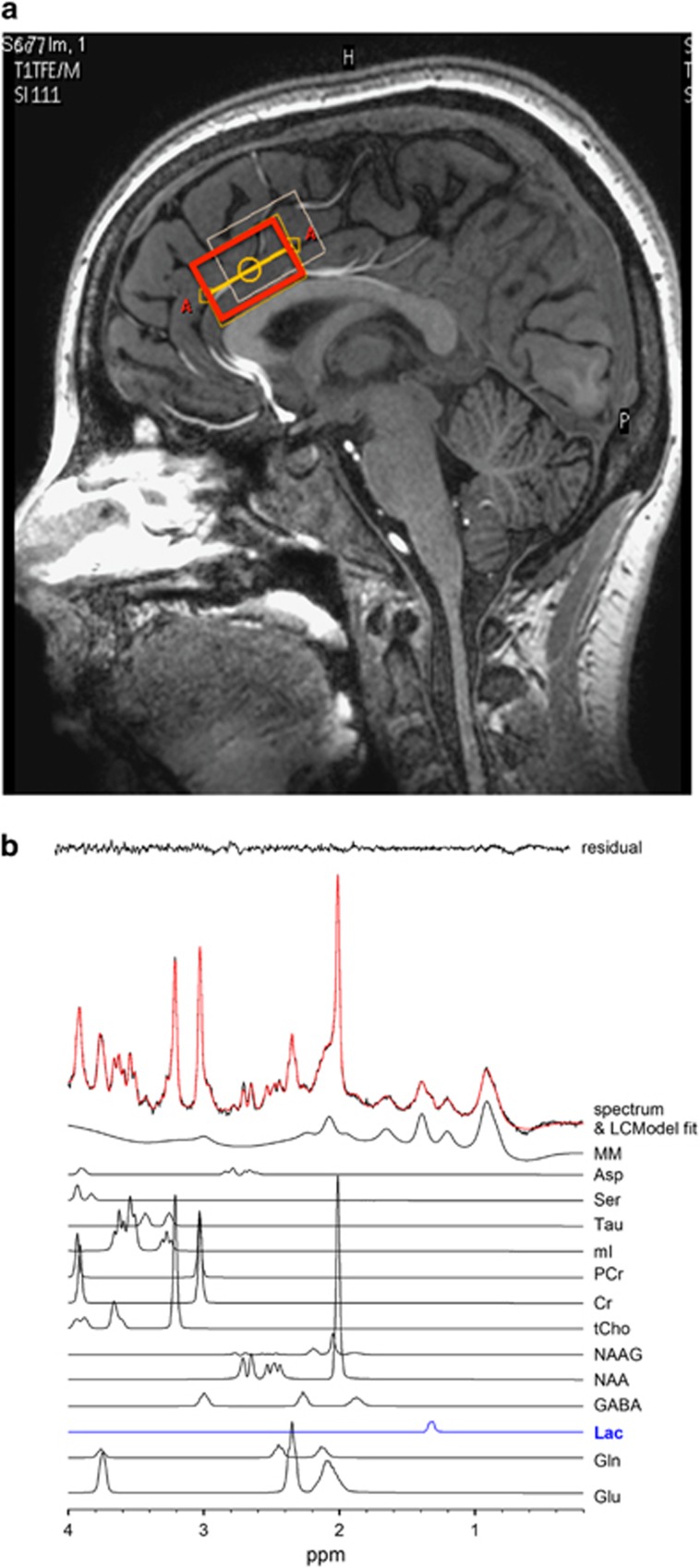
(**a**) Representative anterior cingulate voxel location illustrated in red. (**b**) Representative *in vivo* spectrum (black line), LCModel fit (red line), residual (black line at top) and individual metabolite fits below. Asp, aspartate; Cr, creatine; GABA, gamma-aminobutyric acid; Gln, glutamine; Glu, glutamate; Lac, lactate; MM, macromolecules; ml, myo-inositol; NAA, n-acetylaspartate; NAAG, n-acetylaspartylglutamate; PCr, phosphocreatine; Ser, serine; Tau, taurine; tCho, phosphocholine+glycerophosphocholine.

MR scanning was conducted on a 7 T scanner (Philips ‘Achieva', Best, the Netherlands) equipped with a 32-channel head coil (Nova Medical, Wilmington, MA, USA). Participants were requested to lie still, relax and not fall asleep. Anatomical T1-weighted images were acquired for spectroscopic voxel placement and for CSF correction of the MRS data (sagittal three-dimensional MP-RAGE, 0.8 mm isotropic resolution, TR/TE/TI/FA=4.3 s/1.95 ms/446 ms/7̊, scan time 3 m 40 s). Water-suppressed spectra were acquired from a 30 × 20 × 20 mm voxel positioned in the bilateral anterior cingulate cortex using a STEAM sequence (TE/TM/TR=14/33/3000 ms, 128 averages) and VAPOR water suppression.^17^ The voxel was prescribed on the midsagittal slice and positioned parallel to the genu of the corpus callosum and scalp, and with the anterior boundary of the voxel placed in line with the genu of the corpus callosum. Prior to MRS data collection, shimming was adjusted up to second order using a field-map-based routine, and RF power was optimized on the localized voxel. Two averages were also recorded without water suppression for eddy current correction^18^ automatically performed with Philips post-processing and for quantitation with LCModel.^19^ See Figure 1 for representative voxel placement and corresponding spectra.

Spectra were fitted between 0 and 4.0 p.p.m. using the ‘LCModel' program^19^ using water as an internal reference, and a basis set was simulated in the ‘VESPA' program.^20^ The basis set included alanine (Ala), aspartate (Asp), creatine (Cr), γ-aminobuytric acid (GABA), glucose, glutamate (Glu), glutamine (Gln), glutathione (GSH), glycine (Gly), glycerophosphocholine, lactate (Lac), myo-inositol (mI), n-acetylaspartate (NAA), n-acetylaspartylglutamate (NAAG), phosphocholine (PCh), phosphocreatine (PCr), phosphoroylethanolamine (PE), serine (Ser) scyllo-inositol (sI) and taurine (Tau). The macromolecule basis set provided within LCmodel was used. Metabolite concentrations are expressed in ‘institutional units' (i.u.). All metabolites, except lactate, have negligible concentration levels in the CSF and, therefore, were corrected for the proportion of voxel CSF using the following equation=(metabolite level in institutional units)/(fraction of voxel gray+white matter). Lactate concentrations in the CSF are detectable with MRS and, therefore, were not corrected for CSF.^21, 22^ Lactate fits with percent s.d. Cramer Rao Lower Bounds (%s.d.) ⩽30% were included in statistical analyses. Expanding the criterion of %s.d. (compared with the commonly used value of 20%) is an approach to allow for inclusion of the majority of data while maintaining reasonable quality fitting;^23, 24^ in particular for lower-concentration compounds such as lactate, 20% error corresponds to a very small change in concentration. In addition, choice of too low a Cramer-Rao Lower Bound cutoff can cause bias against low concentration values and non-normal data distribution.^25^ Lactate fits for two control and three patient participants did not meet these criteria, and therefore these participants were eliminated from analysis. The final sample was composed of 29 controls and 27 patients. Statistical analysis including only lactate fits with %s.d. <20% criterion did not change the outcome but are also presented in the Results section.

### Statistical analyses

Demographic variables were analyzed with *χ*^2^-tests for categorical data. Between-group lactate differences were analyzed with analyses of covariance covarying for age and spectroscopic voxel tissue proportions (gray matter, white matter and CSF). Owing to the primary, *a priori* hypothesis regarding lactate, the significance level was set to *P*<0.05. The relationships between lactate, MCCB total score, UPSA total score and psychiatric symptom severity were examined with Pearson's product moment correlations. The significance level was set to *P*<0.0125, Bonferroni-corrected, for the correlational analyses.

The group differences for Gln and Gln/Glu ratio were examined with analyses of covariance covarying for age and spectroscopic voxel tissue proportions (gray matter, white matter and CSF). Owing to the secondary, *a priori* hypothesis based on previous research reporting elevated Gln and Gln/Glu ratio in schizophrenia,^23, 26, 27^ the significance level was set to *P*<0.025, Bonferroni-corrected. If Gln or Gln/Glu reached statistical significance, correlation analyses were conducted in a similar approach to lactate with significance set to *P*<0.0125, Bonferroni-corrected.

## Results

There were no significant differences in age, smoking status or sex between groups. The patient group performed worse on the UPSA and MCCB (all *P*-values<0.05). See Table 1 for means (s.d.'s) for demographic, cognitive and clinical characteristics.

### Lactate

Spectral quality was good with signal-to-noise ratios of 50.3 (6.9) for controls and 46.7 (7.3) for patients and full-width half-maximum (linewidth) of 0.031 (0.005) p.p.m. for controls and 0.032 (0.005) p.p.m. for patients, and there were no statistical differences between groups (both *P*-values>0.05). The mean lactate %s.d.'s were 20.6% (4.4%) for controls and 18.5% (4.0%) for patient participants, and there was no statistical difference between groups (*P*>0.05). Spectroscopic voxel proportion of CSF, gray or white matter did not significantly differ between groups (all *P*-values>0.05, see Table 2).

**Figure 2:**
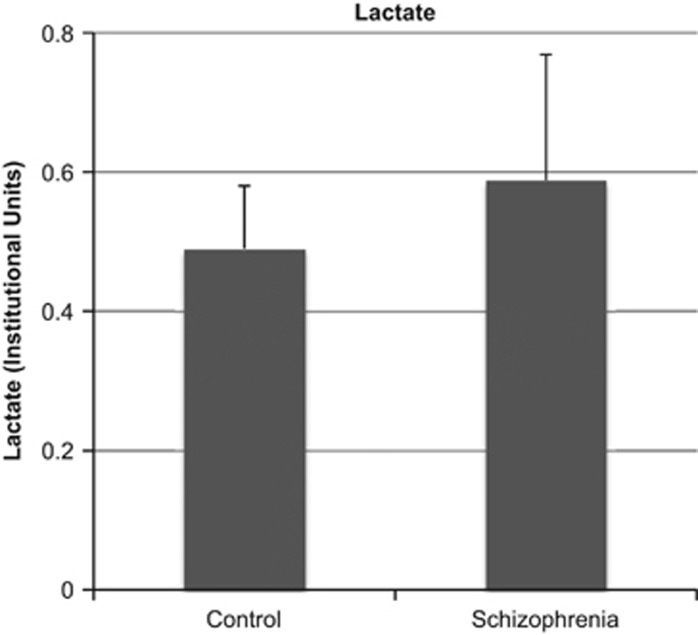
The mean (s.d. bars) lactate levels by diagnostic group. Lactate levels were significantly higher in schizophrenia compared with the control group (*P*<0.05).

Lactate levels were significantly higher in patients with schizophrenia compared with control participants (F=4.2, *P*=0.045). When considering only cases where lactate fit %s.d.<20 (*n*=18 controls, *n*=19 patients), lactate levels were significantly higher in patients compared with controls (F=5.8, *P*=0.022). See Figure 2 for illustration of the mean lactate levels by group.

**Figure 3:**
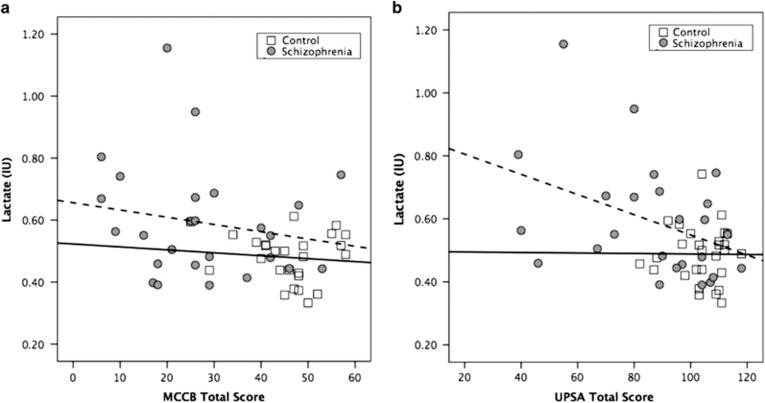
(**a**) A correlation scatter plot illustrating the negative relationship between lactate levels and general cognition function assess with the MCCB (total score). Schizophrenia group, gray circles, dashed trend line with *r*=−0.18, *P*>0.05. Control group, white squares, solid trend line with *r*=−0.10, *P*>0.05. Both groups combined, *r*=−0.36, *P*=0.01. (**b**) A correlation scatter plot illustrating the negative relationship between lactate levels and functional capacity assessed with the UPSA (total score). Schizophrenia group, gray circles, dashed trend line with *r*=−0.40, *P*<0.05. Control group, white squares, solid trend line with *r*=−0.008, *P*>0.05. Both groups combined, *r*=−0.43, *P*=0.001. MCCB, MATRICS Consensus Cognitive Battery; UPSA, University of California, San Diego Performance-Based Skills Assessment.

Lactate was significantly related to MCCB total score (*r*=−0.36, *P*=0.01), such that higher lactate was associated with lower general cognitive function in the total sample. The patient and control groups demonstrated similar relationships (*r*=−0.18, patient versus −0.10, control), and there was no significant difference in the correlation coefficients (*Z*=0.28, *P*=0.78). Lactate was also significantly related to UPSA total score (*r*=−0.43, *P*=0.001), such that higher lactate was associated with lower functional capacity in the total sample. The patient group appeared to drive this association (*r*=−0.40, patient versus −0.008, control), although the statistical difference between correlation coefficients was not significant (*Z*=1.47, *P*=0.14). See Figure 3 for illustration of the relationship between lactate levels, MCCB and UPSA.

The relationship between higher lactate and greater negative symptoms as assessed with the Brief Negative Symptom Scale approached a trend level (*r*=0.34, *P*=0.09). There were no significant or trend relationships between lactate levels, positive symptom severity or CPZ equivalent units^12^ (all *P*-values>0.3).

### Other metabolites

The means (s.d.'s) for the metabolites by group are presented in Table 2. There was a significant difference in Gln/Glu (F=5.7, *P*=0.021) between groups, with patients having higher levels of Gln/Glu compared with control participants. No other metabolites were statistically significantly different between groups. Therefore, only the relationships between Gln/Glu and clinical and cognitive measures were explored.

Gln/Glu was significantly related to MCCB (*r*=−0.4, *P*=0.004) and UPSA total scores (*r*=−0.45, *P*=0.001) in the total sample. The patient and control groups demonstrated similar relationships for the MCCB (*r*=−0.29 versus *r*=0.24) and the difference between the correlation coefficients was not significant (*Z*=0.18, *P*=0.86). The patient group had a stronger UPSA–Gln/Glu association (*r*=−0.52 versus −0.19 for controls), although the statistical difference between correlation coefficients was not significant (*Z*=1.36, *P*=0.17). Gln/Glu was not significantly related to Brief Negative Symptom Scale, Brief Psychiatric Rating Scale-positive symptom or CPZ (all *P*-values>0.05).

### Antipsychotic medication status

There were no significant lactate, Gln, Glu, Gln/Glu differences between the off and on medication groups (*P*-values range from 0.2 to 0.96). There were also no significant lactate, Gln, Glu and Gln/Glu differences between those treated and those not treated with clozapine (*P*-values range from 0.1 to 0.8).

## Discussion

To the best of our knowledge, this study reports for the first time elevated *in vivo* brain lactate levels in participants with schizophrenia compared with healthy comparison subjects. Higher levels of lactate in the anterior cingulate cortex were related to poorer general cognitive function and poorer functional capacity. Anterior cingulate Gln/Glu ratios were also higher in participants with schizophrenia, which is consistent with previous reports.^23, 26, 27^ Similar to lactate, higher anterior cingulate Gln/Glu was also related to poorer general cognitive function and poorer functional capacity.

Higher levels of frontal lactate in schizophrenia likely reflect altered bioenergetics; however, the exact mechanism remains unknown. Possibilities include altered glucose metabolism and impaired mitochondrial oxidation. One interpretation is that there is impaired mitochondrial energy metabolism causing a shift to greater cytosolic glycolysis, and this could generate elevated brain lactate. A recent post-mortem study reported that reduced mitochondrial density in the anterior cingulate in schizophrenia^4^ provides support for this interpretation. Hence, our results of elevated lactate support this interpretation and further complement a recent ^31^P magnetization transfer MRS study^7^ and a CSF study.^6^

It is also well known that elevations of lactate are observed in the brain in patients with mitochondrial diseases with cerebral involvement,^28^ but that elevated brain lactate can also occur as the result of other pathological mechanisms also, for example, in hypoxia/ischemia, tumors and inflammation.^29, 30, 31, 32^ Increased lactate has also been reported in other psychiatric disorders, such as bipolar disease.^33^ It should also be noted that CSF tends to have a more prominent lactate signal than brain tissue in some pathological conditions,^22^ which may be because of a combination of higher concentration and/or longer T_2_ relaxation time in CSF fluid. Therefore, when examining small lactate concentrations in the brain, it is important to make sure that partial volume with CSF is not driving the between-group differences; in the current study, there were no significant differences in voxel CSF content in subjects with schizophrenia and controls, and covarying for voxel CSF proportion did not change the results.

Oxidative stress is another possible explanation for elevated frontal lactate in schizophrenia. One preclinical study reported elevated lactate levels in a schizophrenia mouse model of chronic GSH deficit induced by knockout of a GSH-synthesizing enzyme, Glu–cysteine ligase-modulatory subunit.^5^ Interestingly, elevated levels of Gln and Gln/Glu were also observed in this knockout mouse, similar to the results of the current study. The altered metabolites may be due to oxidative stress-induced mitochondrial dysfunction^34^ or vice versa—that is, mitochondrial dysfunction induced overproduction of reactive oxygen species leading to oxidative stress.^35^ The current study did not find lower GSH in the schizophrenia group, in contrast to this preclinical study. However, this is not entirely surprising as GSH levels vary depending upon Glu–cysteine ligase catalytic polymorphism, with high-risk genotypes exhibiting lower levels of GSH in the medial frontal cortex encompassing the anterior cingulate.^36^

Lactate levels were related to general cognitive function (MCCB) and functional capacity (UPSA), with higher levels associated with poorer performance. The relationships for general cognitive function were similar in both groups, but for functional capacity the relationship was stronger in the patient group, although not significantly different. It is reasonable to speculate that higher lactate levels reflect mitochondrial dysfunction causing altered bioenergetics that could negatively have an impact on neurotransmission and synaptic plasticity.^1, 37^ These altered mechanisms likely contribute to cognitive/functional capacity impairments. Mitochondrial dysfunction has been linked to diseases characterized by cognitive and functional impairments such as Alzheimer's disease,^38^ other forms of dementia,^39^ Parkinson's disease^40^ and diabetes.^41^ Moreover, cognitive dysfunction is frequently observed in mitochondrial disorders.^42^ Interventions targeted to enhance mitochondrial function may prove beneficial for cognition and functional capacity in schizophrenia.

Several study limitations are worth mentioning. This study did not employ a spectroscopic technique specifically tailored for lactate detection, such as spectral editing.^43, 44^ However, several 7 T studies focused on lactate detection using a similar spectroscopic approach as the current study,^45, 46^ and the lactate signal, although small, was reliably detected with reasonable Cramer-Rao Lower Bound values in nearly all subjects in the study. There is a possibility that macromolecules, broad signals that underlie metabolites in short TE spectra, could have an impact on the results. The LCmodel basis set of macromolecules was used for spectral fitting; however, future studies should use individually acquired macromolecule spectra for spectral fitting or utilize acquisition techniques that suppress macromolecule signals. As common to most studies of schizophrenia, the participants with schizophrenia were treated with antipsychotic medication, which could affect the findings. One study reported that increased frontal cortical lactate concentrations in rats administered clozapine or haloperidol for 28 days,^3^ but another study found no effect with 6 months of haloperidol administration.^47^ No significant relationship between CPZ daily units and lactate levels was found, which is consistent with previous post-mortem lactate^3^ and MRS^7^ work, but could also be influenced by ceiling effects. Furthermore, Regenold *et al.*^6^ reported lower CSF lactate in patients taking antipsychotics than patients not taking antipsychotic medication, which we did not find in this study. Results are mixed with respect to Gln/Glu and antipsychotic medication, with some studies reporting elevated glutamatergic metabolites in patients off antipsychotic medication,^48, 49, 50, 51^ on antipsychotic medication^23, 52, 53^ and in treatment-resistant patients treated with antipsychotic medication.^54, 55^ Similar to lactate, there was no significant relationship between Gln/Glu levels and CPZ daily units, no difference between on and off medication groups and no difference with those taking clozapine. Finally, we did not conduct urine drug screens or assess mood or physical activity before the MR scan, or rigorously control for the resting state during the MR scan, which could have an impact on results.

The results of this study support the hypothesis that brain bioenergetics are altered in schizophrenia, reflecting inefficient or diminished aerobic metabolism and a shift toward anaerobic metabolism. Elevated lactate may prove to be a useful biomarker of cognitive and functional capacity in schizophrenia. Interventions to promote more efficient mitochondrial energy metabolism may prove useful for enhancing cognition and alleviating functional impairments in schizophrenia.

## Figures and Tables

**Table 1:** Participant characteristics (mean (s.d.))

	*Schizophrenia (*n*=27)*	*Control (*n*=29)*	*Statistics (*t *or* χ^*2*^)	P
Gender (male/female)	17/10	14/15	1.2	0.27
Age (years)	34.4 (13.1)	29.7 (9.4)	1.5	0.13
Smoking status (no/yes)	20/7	23/6	0.22	0.64
MCCB total score	27.8 (14.3)	45.9 (8.2)	5.6	<0.001^a^
UPSA total score	86.7 (23.0)	104.0 (8.9)	3.6	0.001^a^
BPRS-negative symptoms	6.4 (2.0)	n/a	n/a	n/a
BPRS-positive symptoms	8.3 (3.4)	n/a	n/a	n/a
BPRS total score	38.3 (7.4)	n/a	n/a	n/a
Years ill	13.1 (12.1)	n/a	n/a	n/a
Chlorpromazine equivalent	381.1 (346.24)	n/a	n/a	n/a
				
*Antipsychotic medication (n)*				
Navane	1			
Clozapine	7			
Olanzapine	2			
Abilify	3			
Risperdal	5			
Lurasidone	1			
Paliperidone	1			
Olanzapine/quetiapine	1			
Olanzapine/risperidal	1			
Off medication	5			

Abbreviations: BPRS, Brief Psychiatric Rating Scale; MCCB, MATRICS Consensus Cognitive Battery; n/a, not applicable; UPSA, University of California, San Diego Performance-Based Skills Assessment.

a Statistically significant.

**Table 2:** Anterior cingulate metabolite and voxel tissue proportion means (s.d.) in the schizophrenia and control groups

	*Schizophrenia (n=27)*	*Control (n=29)*	*Statistic (F)*	*P*
*MRS metabolites (IU)*				
Glutamate	7.9 (0.85)	8.1 (0.67)	0.23	0.63
Glutamine	1.9 (0.28)	1.8 (0.36)	5.1	0.028
Glutamine/glutamate	0.25 (0.04)	0.22 (0.05)	5.7	0.021^a^
Lactate	0.59 (0.18)	0.49 (0.09)	4.4	0.045^a^
GABA	1.6 (0.21)	1.7 (0.18)	1.1	0.31
n-acetylaspartate	7.6 (0.67)	7.8 (0.44)	2.2	0.14
NAAG	0.64 (0.14)	0.67 (0.15)	0.23	0.64
Glycerophosphocholine+phosphocholine	1.6 (0.20)	1.5 (0.16)	1.3	0.27
Creatine	3.6 (0.40)	3.5 (0.43)	0.39	0.53
Phosphocreatine	2.3 (0.35)	2.4 (0.30)	0.32	0.57
Myo-inositol	5.8 (0.64)	5.8 (0.42)	0.23	0.63
Taurine	1.3 (0.27)	1.3 (0.21)	0.46	0.50
Serine	1.4 (0.34)	1.4 (0.47)	1.02	0.32
Aspartate	1.4 (0.22)	1.5 (0.16)	0.99	0.33
Glutathione	1.5 (0.16)	1.5 (0.11)	0.16	0.69
				
*MRS voxel (%)*				
CSF	11.0 (3.6)	10.6 (3.9)	-0.42	0.68
White matter	19.2 (6.1)	18.1 (3.7)	-0.83	0.41
Gray matter	69.8 (5.4)	71.3 (3.6)	1.23	0.22

Abbreviations: CSF, cerebrospinal fluid; GABA, gamma-aminobutyric acid; IU, institutional unit; MRS, magnetic resonance spectroscopy; NAAG, n-acetylaspartylglutamate.

a Statistically significant.
